# Determining the frequency of thyroid involvement in chest CT scans of COVID-19 patients and its correlation with the severity of lung involvement and survival of patients in 2020

**DOI:** 10.3389/fendo.2024.1345008

**Published:** 2024-07-09

**Authors:** Fatemeh Yarmahmoodi, Shoayb Samimi, Banafasheh Zeinali-Rafsanjani, Seyed Mostajab Razavinejad, Mahdi Saeedi-Moghadam

**Affiliations:** ^1^ Medical Imaging Research Center, Shiraz University of Medical Sciences, Shiraz, Fars, Iran; ^2^ Department of Radiology, Shiraz University of Medical Sciences, Shiraz, Iran; ^3^ Neonatal Research Center, Shiraz University of Medical Sciences, Shiraz, Fars, Iran

**Keywords:** COVID-19, involvement of the thyroid gland, chest CT scan, severity of pulmonary involvement, thyroid function

## Abstract

**Introduction:**

This study aimed to determine the frequency of thyroid gland involvement in chest CT scans of patients with COVID-19 admitted to university-affiliated hospitals and assess its relationship with the severity of lung involvement and patient survival in 2020.

**Material and methods:**

In this retrospective cross-sectional study, 1000 PCR-positive patients with COVID-19 who were referred to University-affiliated Hospital in 2020 and had chest CT performed within 72 hours of admission to the hospital were examined. The data was collected by patient file information and CT findings recorded in the PACS system, including thyroid involvement, the severity of lung involvement, and findings related to the death and recovery of patients.

**Results:**

The mean age of the examined patients was 56 years. 525 people (52.5%) were men, and 475 (47.5%) were women. 14% had severe pulmonary involvement, and 9.3% had very severe involvement. Moreover, 15.9 percent of them had deceased. 19.7% had focal thyroid involvement, 14% had diffuse involvement, and 66.3% were healthy subjects. Male gender and older age showed a significant relationship with thyroid gland involvement. The severity of lung involvement, the death rate in patients, and hospitalization in ICU were also significantly related to thyroid gland involvement in patients with COVID.

**Discussion and conclusion:**

This study highlights the importance of considering thyroid-gland involvement in the comprehensive management of COVID-19 patients. Routine screening and monitoring of thyroid-function may facilitate earlier detection and appropriate management of thyroid-related complications, potentially improving clinical outcomes. This study suggests that in COVID-19 infection the monitoring of thyroid function is prudent, particularly in cases of more serious disease.

## Highlights

- The severity of lung involvement had a significant relationship with thyroid involvement in patients with COVID-19.
- Patients with thyroid involvement (focal and diffuse) had a higher severity of lung involvement; most showed severe and very severe lung involvement.
- Thyroid function monitoring should be strengthened in patients with COVID-19.

## Introduction

Severe Acute Respiratory Syndrome (SARS) is a human-animal pathogen that emerged in December 2019 and caused the pandemic of COVID-19 (1). This virus is classified as one of the RNA viruses belonging to the coronavirus family (2). The information about the characteristics and clinical results of patients with severe disease infected with COVID-19 is still essential to reduce the mortality of infected people (3).

Severe cases of Covid-19 cause pneumonia, respiratory failure, and death due to multiple organ failure. In contrast, in mild cases, the usual respiratory tract infection symptoms may not be present (4). In addition to the respiratory system, COVID-19 can affect several other organs and systems (5), including the endocrine system (6, 7), with possible short-term and long-term consequences (8). The latest research results indicate that COVID-19 can damage the thyroid gland. People who suffer from thyroid gland inflammation during the severe type of COVID-19 may still suffer from subacute thyroiditis several months later (9).

The pituitary-thyroid axis should be considered a sensitive target of SARS-CoV-2, and a direct or indirect pituitary injury has been described as a determinant of secondary hypothyroidism (10). In line with these suggestions, thyroid dysfunction can be observed during and after COVID-19 infection, so it is expected that some new or recurrent thyroid dysfunction can be attributed to recent SARS-CoV-2 infection., pre-existing or new-onset thyroid hormone imbalance, such as low T3 syndrome, can be associated with disease severity in COVID-19 (11).

It seems that the virus that causes COVID-19 disease damages the thyroid gland. This gland produces two substances called ACE2 and intramembrane serine protease in high amounts. These two substances allow the SARS-CoV-2 virus to infect human cells. Research experts agree that such patients should be monitored because thyroid function disorders may occur in these people in the coming months. It has also been proposed that these people should perform thyroid tests every 6 months for a year (12). COVID-19 induces hyperactivity of the immune system, especially interleukin-6 (IL-6), which can lead to thyroid dysfunction due to the disruption of deiodase and thyroid transport nutrients (13, 14). Hence, evaluating thyroid function abnormalities is essential in COVID-19 (15, 16). Some studies revealed that the thyroid gland could be the target of SARS-CoV-2 (17, 18).

Müller et al. showed that classic subacute thyroiditis usually affects women, while the thyroiditis induced by COVID-19 affects mostly men (19). Classic subacute thyroiditis has also been described after mild infection with SARS-CoV-2 and is associated with typical neck pain and is more common in women. Previous studies showed that these individuals typically had low or suppressed serum thyroid-stimulating hormones (TSH), with and without increasing the free thyroxine concentration, indicating thyrotoxicosis. In patients with non-thyroid disease syndrome, normal or low serum concentration of thyroid-stimulating hormone and low concentration of triiodothyronine are usually associated with a low concentration of thyroxine (20, 21). However, Müller et al. declared that in patients with COVID-19, low thyroid-stimulating hormone and triiodothyronine concentrations were associated with normal or increased thyroxine concentrations (19).

Müller also suggests routine evaluation of thyroid function in patients with COVID-19 requiring intensive care, as they often present with thyrotoxicosis due to subacute thyroiditis related to SARS-CoV-2. The normal thyroid gland has a high attenuation (80-100 HU) because it concentrates iodine approximately 100 times more than the serum. In subacute thyroiditis, a diffusely swollen thyroid gland is seen, with low attenuation corresponding to 40 HU. Although some studies have described the sonographic features associated with subacute granulomatous thyroiditis, few reported the CT imaging features and MR associated with subacute granulomatous thyroiditis (22).

Examining the thyroid function in patients with COVID-19 may help to discover the pathogenesis of SARS-CoV-2 and provide practical information for clinical practice. The present study aims to determine the frequency of thyroid gland involvement in chest CT scans of patients with COVID-19 admitted to university-affiliated hospitals and assess its relationship with the severity of lung involvement and patient survival in 2020.

## Materials and methods

This was a retrospective cohort study. The frequency of thyroid gland involvement in chest CT scans of patients with COVID-19 admitted to University-affiliated hospitals and its relationship with the severity of lung involvement and patient survival in 2020 was investigated.

Patients infected with COVID-19 from the beginning to the end of 2020, whose disease was diagnosed using PCR tests or CT scans and referred to university-affiliated hospitals, were included in the study. Patients with clinical symptoms of COVID-19 without a positive PCR test, low-quality CT scans, or incomplete information were excluded.

It is worth noting that, non-contrast chest CT scans which were in routine diagnostic programs was used to evaluate thyroid gland. As mentioned previously, thyroiditis usually exhibits hypodensity on non-contrast CT scans, which can be attributed to the reduced concentration of iodine.

The data was collected using a checklist prepared from the patient’s records. This checklist had three parts. The first part was about the patient’s demographic information, history of chronic disease, and duration of hospitalization. The second part was about CT scan findings of thyroid involvement and the severity of lung involvement. The third part was the data related to the death and recovery of the patients.

A researcher referred to the archive section of hospitals and extracted the list of patients who met the inclusion criteria. Then the CT scan of included patients was assessed on PACS to assess the CT findings and record them in the checklist. The patient’s condition at discharge was assessed using the hospital information system.

Statistical analysis was performed using SPSS version 24. Descriptive data were analyzed using the mean and standard deviation. Independent t-tests were used to analyze the quantitative data. The significance level in this study was considered to be 0.05.

## Results

One thousand patients with COVID-19 were included, with a mean age of 56 ± 17.71 years and an age range of 16 to 97 years old. 525 cases (52.5%) were men with a mean age of 56.40 ± 18.17, and 475 cases (47.5%) were women with a mean age of 55.56 ± 17.18. This difference was not statistically significant (P=0.45). Assessment of the frequency distribution of patients showed that the highest frequency was related to the age group of 70 years and older (24.2%), and the lowest frequency was related to the age group less than 30 (6.8%).

**Figure 1:**
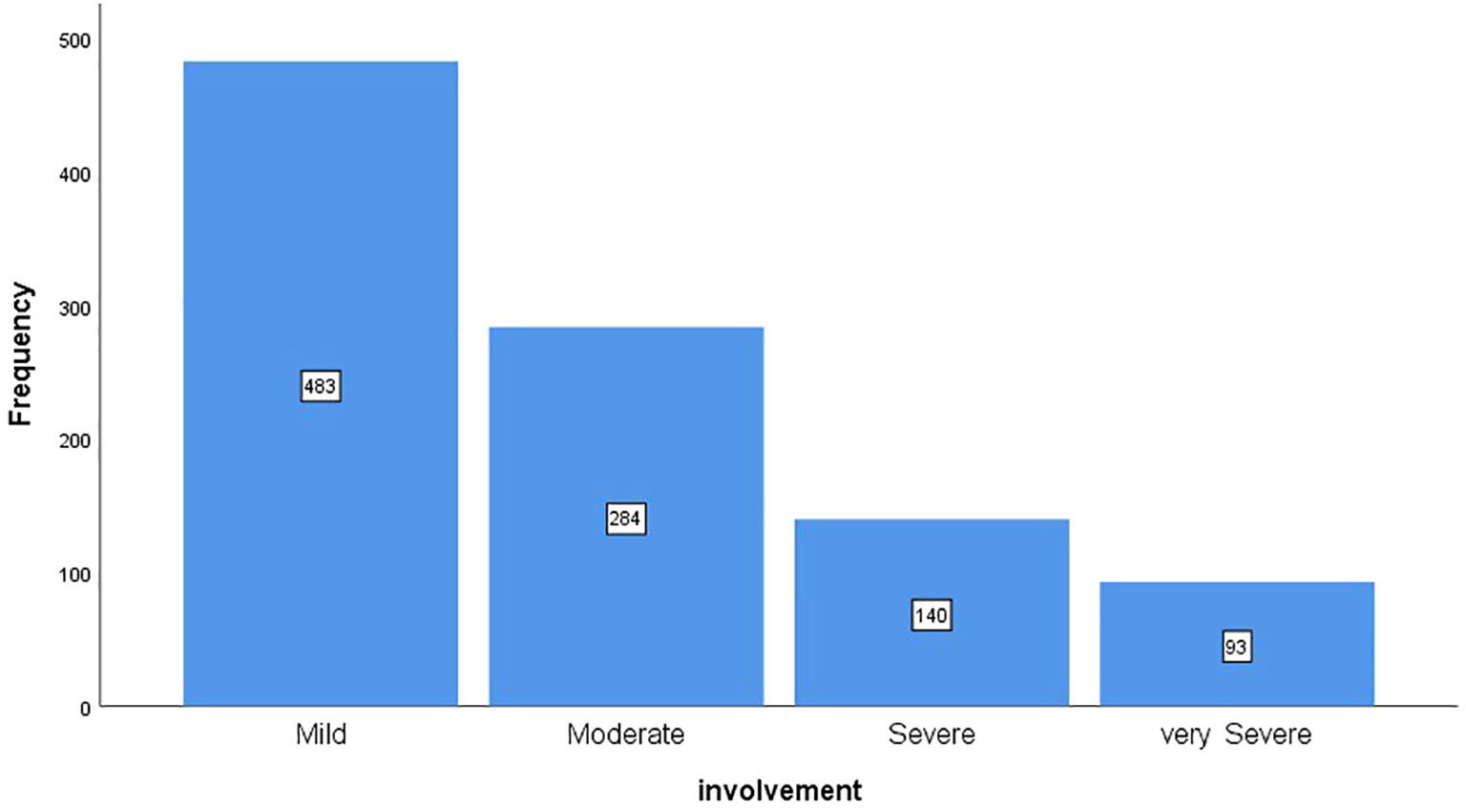
The frequency distribution of patients based on the severity of lung involvement.

**Figure 1** shows the frequency distribution of patients based on the severity of lung involvement. According to the chart, 48.3% of patients had mild involvement, 28.4% had moderate involvement, 14% had severe involvement, and 9.3% had very severe involvement.

The frequency distribution of patients based on recovery and death showed that 84.1% had recovered and 15.9% had died. 162 patients (16.2%) were admitted to ICU. Among these 162 patients, 63 (38.9%) had recovered, and 99 (61.1%) had died.

**Figure 2:**
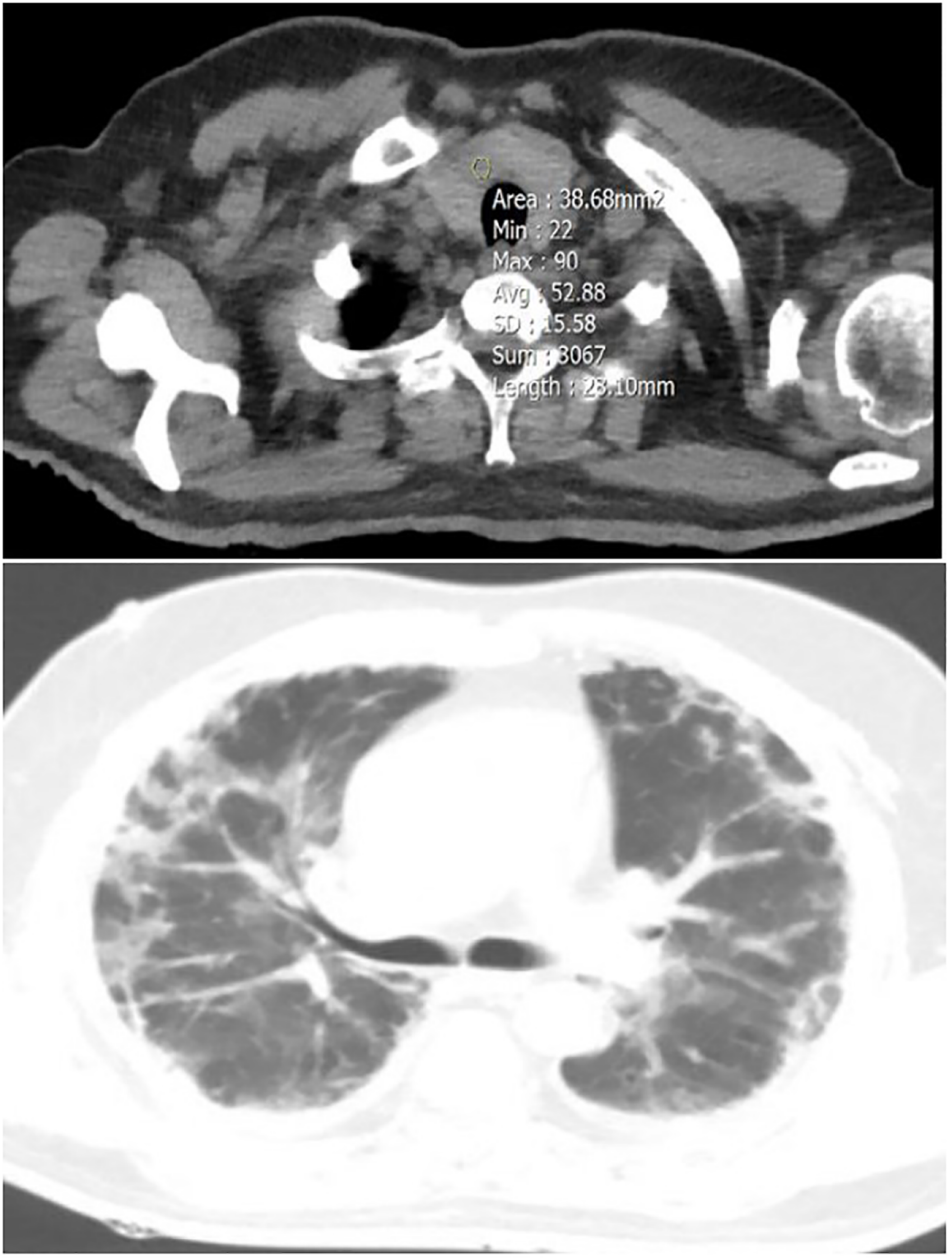
A 54-year-old male patient with focal thyroid involvement (mean HU=52.88) (upper image), and mild Lung involvement (lower image).

**Figure 3:**
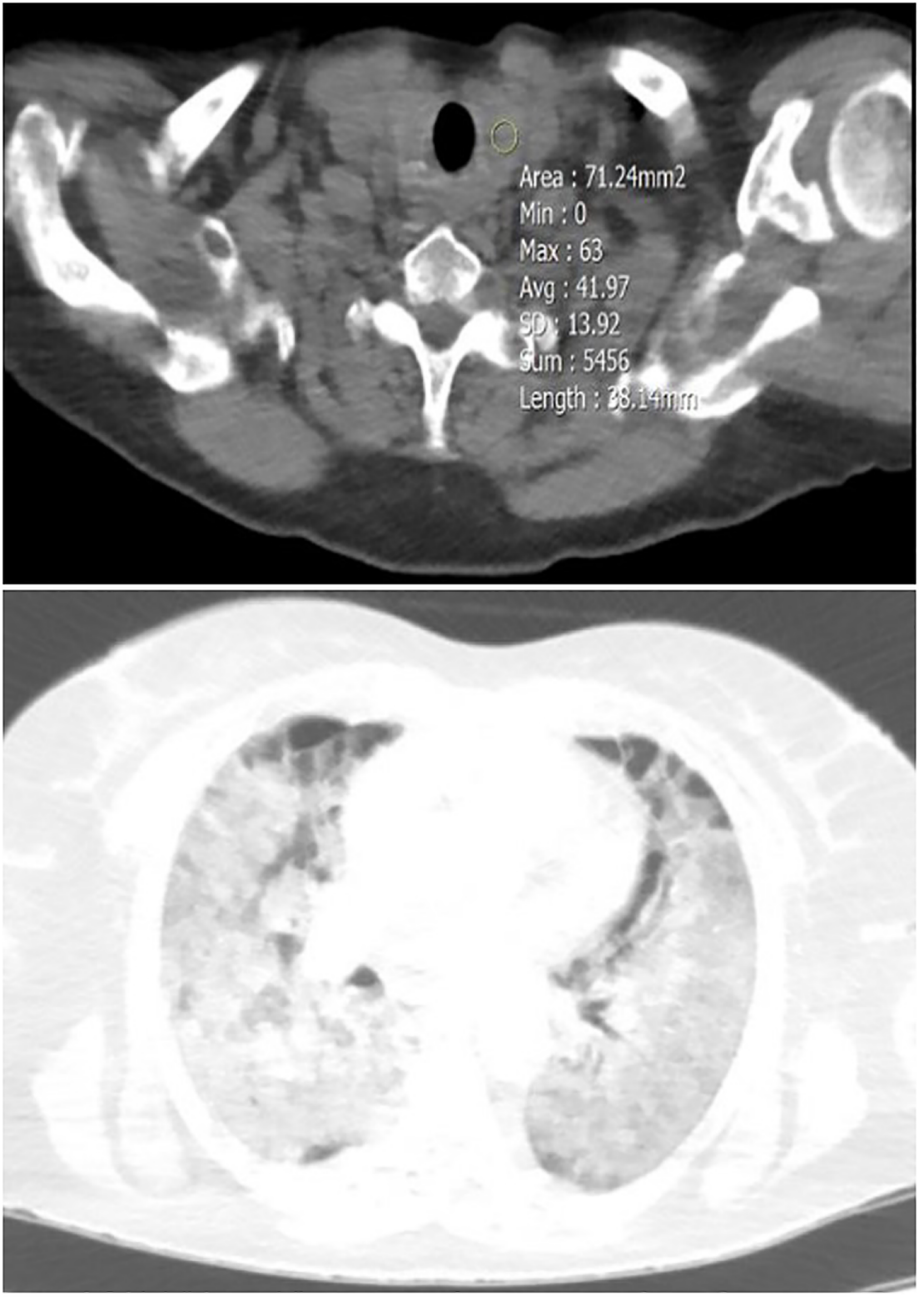
A 40-year-old female patient with diffuse thyroid involvement (mean HU=41.97) (upper image), and severe Lung involvement (lower image).

Assessment of the frequency distribution of patients based on thyroid gland involvement showed 19.7% of focal involvement, 14% of diffuse involvement, and 66.3% of healthy subjects. **Table 1** shows the frequency distribution of thyroid gland involvement based on patient age and sex. The focal and diffuse involvement frequency was higher in male patients than in female patients. However, this difference was not statistically significant. The frequency of focal and diffuse involvement in the age groups of 60-70 and above 70 years was significantly higher than in other age groups. The average age of patients with focal involvement was 59.74 ± 16.87; in patients with diffuse involvement, it was 62.60 ± 15.11, and in patients without involvement, it was 53.50 ± 17.92 years, which was statistically significant (P<0.001) (**Figures 2**, **3**).

**Table 1:** The frequency distribution of thyroid gland involvement based on patient age and sex.

Variable		Focal	Diffuse	Normal	p
**Sex**	Male	107(54.3%)	82(58.6%)	336(50.7%)	0.20
	Female	90(45.7%)	58(41.4%)	327(49.3%)	
**Age**	<30	8(4.1%)	6(4.3%)	54(8.1%)	<0.001
	30-40	22(11.2%)	4(2.9%)	120(18.1%)	
	40-50	29(14.7%)	17(12.1%)	114(17.2%)	
	50-60	29(14.7%)	20(14.3%)	110(16.6%)	
	60-70	42(21.3%)	50(35.7%)	133(20.1%)	
	>=70	67(34.0%)	43(30.7%)	132(19.9%)	

**Table 2** shows the frequency distribution of thyroid gland involvement based on the severity of lung involvement in patients. The majority of patients with focal thyroid involvement had moderate lung involvement. On the other hand, most patients with diffuse involvement had severe and very severe lung involvement. Healthy patients, on the other hand, exhibited mild lung involvement, which was found to be statistically significant (P<0.001).

**Table 2:** The frequency distribution of thyroid gland involvement based on patients’ lung involvement severity.

Thyroid involvement Lung involvement		Focal	Diffuse	Normal	p
**Severity**	Mild	70(35.5%)	7(5.0%)	406(61.2%)	<0.001
	Moderate	80(40.6%)	24(17.1%)	180(27.1%)	
	Severe	36(18.3%)	46(32.9%)	58(8.7%)	
	very Severe	11(5.6%)	63(45.0%)	19(2.9%)	

**Table 3** presents the frequency distribution of pulmonary thyroid gland involvement based on recovery status, death, and the need for ICU admission in patients. The death rate was higher in patients with diffuse involvement compared to those with focal involvement or no thyroid involvement. This difference was statistically significant (P<0.001). Additionally, the frequency of ICU hospitalization was significantly higher in patients with both focal and diffuse involvement compared to those without thyroid involvement.

**Table 3:** The frequency distribution of pulmonary thyroid gland involvement based on recovery status and death in patients and the need for ICU admission.

Thyroid involvement Variables		Focal	Diffuse	Normal	p
**Patient status at discharge**	Recovery	16 0(81.2%)	65 (46.4%)	616 (92.9%)	<0.001
	Death	37 (18.8%)	75 (53.6%)	47 (7.1%)	
**Admission in ICU**	Yes	44 (22.8%)	69 (49.3%)	48 (7.2%)	<0.001
	No	152 (77.2)%	71 (50.7%)	615 (92.8%)	

## Discussion

The pathogenesis of COVID-19 may affect several human organ systems in different ways. In particular, severe COVID-19 is characterized by organ dysfunction, hypercytokinemia, and lymphopenia (23). There are many reports of thyroid disorders in patients with acute COVID-19 infection (24). There may be multiple effects of thyroid dysfunction on the risk and severity of COVID-19. Based on this, the present study investigated the frequency of thyroid gland involvement in chest CT scans of patients with COVID-19 admitted to Shahid Faqihi Hospital in Shiraz and its relationship with the severity of lung involvement and patient survival in 2020.

Chest CT imaging plays a vital role in the diagnosis and dynamic assessment of COVID-19. The typical imaging features of multiple consolidations and/or GGOs in patients with COVID-19 pneumonia have been described in detail in previous reports (25, 26). The severity of COVID-19 affects the treatment options, and the chest CT findings, which detect the severity of the lung involvement of the disease, are relevant for the treatment and management of the COVID-19 disease. According to the results of the present study, the severity of lung involvement in most patients (48.3%) was mild. 28.4% had moderate involvement, 14% had severe involvement, and 9.3% had very severe involvement.

Similar findings were observed by Hafez and Francon et al. (27, 28). The severity of the disease in CT was classified as mild, moderate, or severe using lung opacity and CT-SS calculation (29). Most patients had moderate (52.5%) and severe (27.5%) CT-SS. In Alian et al.’s study, most patients showed moderate lung involvement in the CT scan findings (30). According to recent studies, chest CT scans help in early diagnosis of COVID-19 and in evaluating the severity of pulmonary involvement (31, 32). In addition, it can provide prognostic information related to the progression to severe stage (32). It is also considered a valuable tool for identifying patients who need invasive treatments instead of routine treatment (33). So far, the degree of involvement and the rapid progress of CT findings are the most essential clinical criteria for the severity of COVID-19 (34).

According to the evidence, the 2-CoV-SARS virus can affect a wide range of organs since the penetration of this virus into the host cells is done with the help of the ACE2 receptor. On the other hand, the expression of the ACE2 receptor gene is high in the thyroid; this gland can be one of the potential targets of 2-CoV-SARS. The possible effect of COVID-19 on the thyroid is not limited to the direct penetration of the virus. However, it may involve this hypothalamus-pituitary axis disease, which closely interacts with the thyroid gland’s functioning (35). According to the results of our study, regarding thyroid involvement, 19.7% of patients had focal involvement, 14% had diffuse involvement, and 66.3% were healthy.

The survey found that the highest prevalence of thyroid disorders in patients with COVID-19 was observed in the Chinese report at 64% (36), and the lowest prevalence of thyroid dysfunction was observed in the Chinese report with a prevalence of 1.2% (37). The results of a review study by Nouri et al. (38) showed that the prevalence of thyroid dysfunction among 9707 patients with COVID-19 was 15%. Muller et al. observed thyrotoxicosis in 15.3% of patients with COVID-19 compared to only 1.3% of controls in their study (19). Another retrospective study of 287 non-critical COVID-19 patients by Lania et al. found that 20.2% had thyrotoxicosis and that thyroid function assessed during hospitalization was associated with concentrations of several inflammatory markers (39).

There was extensive follicular damage, with large numbers of exfoliated cells in the follicle in thyroid glands. The follicular structure was prominently affected and showed follicular collapse (40). In the first report of subacute thyroiditis (SAT) associated with COVID-19 infection, diffuse hypoechoic areas were reported on thyroid ultrasound, and changes in FT3, FT4, TSH, and TgAb (41). Subsequently, other studies have shown changes in thyroid ultrasound of COVID-19 patients with SAT, including bilateral hypoechoic areas (42, 43), heterogeneity in the parenchyma (44), relative diffuse reduction of vascularity (45) and increased vascularity (42), and inflammation (39). created. Computed tomography of the chest also showed that COVID-19 patients had altered thyroid tissue density during their infectious state compared to prior infection. Similarly, a case report of thyroid storm associated with SARS-CoV-2 also identified ultrasound changes in the thyroid. The 25-year-old female patient presented with exophthalmos, tachycardia, an enlarged diffuse goiter with bruit, and a fine tremor. Laboratory results showed deficient levels of TSH (TSH<0.01 mIU/L) and high levels of FT4 (5.34 ng/dL) and TT3 (654 ng/dL). Thyroid ultrasound showed heterogeneous echotexture with increased vessels (46).

The results of our study showed that the severity of lung involvement had a significant relationship with thyroid involvement in patients with COVID-19. The results showed that patients with thyroid involvement (focal and diffuse) had a higher severity of lung involvement; most showed severe and very severe lung involvement.

In the study of Nouri et al. (38), thyroid dysfunction was associated with the severity of COVID-19, as its prevalence was 6.2% in mild to moderate cases versus 20.8% in patients with severe COVID-19. These results were consistent with the results of our study. Consistent with the results of the present study, in a study comparing patients with mild or severe COVID-19 pneumonia, none of the patients hospitalized with mild pneumonia had hypothyroidism, whereas 3.2% of those with pneumonia had hypothyroidism had severe hypothyroidism (2.4% overt and 0.8% subclinical) (47). In a study of 433 patients hospitalized with COVID-19, hypothyroidism was present and treated in 43 patients (9.9%) and was significantly associated with severe COVID-19 (48). A recent prospective study (49) comparing 125 patients with mild COVID-19 pneumonia to 125 patients with severe COVID-19 pneumonia showed that 13% of those with severe pneumonia had hyperthyroidism (6.4% overt and 5.6% subclinical). In contrast, the mild pneumonia group had a lower prevalence (1.6% overt and 4.8% subclinical). One study suggested that patients with SAT should be tested for SARS-CoV-2 infection and showed that young people can develop mild forms of COVID-19 and SAT without showing any signs of chronic thyroid dysfunction (50). Chen et al. showed that the serum concentration of TSH and total T3 in patients with COVID-19 was significantly lower than in the control group (51).

Because of the imbalance in the immune system, patients with autoimmune thyroid disorders may have more severe COVID-19 than healthy individuals due to higher baseline serum concentrations of IL-6 and TNF-α. However, in susceptible patients, SARS-CoV-2 may impair immune tolerance, leading to immune-mediated thyroiditis, exacerbation of pre-existing thyroid disease, or recurrence of pre-existing thyroid disorder (9). The data showed that abnormal thyroid function may occur during and after the recovery phase of COVID-19. Although the cellular and molecular mechanisms are not fully understood, evidence suggests that the “cytokine storm” is an important mediator. Likely, indirect mechanisms (such as increased serum cytokines and immune cells) are responsible for most of the effects observed in the entire HPT axis.

On the other hand, some authors have also suggested that thyroid cells can be directly infected by SARS-CoV-2. ACE2 mRNA has been consistently shown in multiple data sets to be expressed in both human thyroid tissues and primary cultured cells, suggesting that the thyroid can be vulnerable to direct viral infection and its cytopathic effects. Hence, altered thyroid function during COVID-19 is more likely to result from proinflammatory signals and impaired central control than direct infection of follicular cells by SARS-CoV-2 (12, 52). It should be explained that since the entry of the COVID-19 virus into the body causes many immune-inflammatory reactions, these reactions can lead to cytokine storms and autoimmune thyroid diseases. It is not yet clear how susceptible people with thyroid function disorders are to contracting Covid-19 and whether they will suffer more severe consequences if they do.

It should be noted that after more than two years have passed since the beginning of the COVID-19 pandemic, there are still many unknowns about this disease. Knowing the effect of COVID-19 on the thyroid requires extensive pathological studies to determine the direct or indirect role of 2-CoV-SARS on the thyroid gland and its function. On the other hand, with the spread of general vaccination against the coronavirus, studying the possible effect of different vaccines on the thyroid is one of the daily needs of the medical and health communities.

The two outcomes investigated in our study were ICU admission and patient mortality. According to the results of our study, the percentage of patients with thyroid involvement (focal and diffuse) hospitalized in the ICU was significantly higher than those without thyroid involvement. Additionally, the frequency of death in patients with thyroid involvement (focal and diffuse) was significantly higher than in patients without thyroid involvement.

Not many studies have evaluated the postmortem thyroid of patients who died of Covid-19. Of these, some found no significant damage to thyroid follicular cells (18), while others reported chronic thyroid inflammation, follicular epithelial cell disruption, or interstitial lymphocytic infiltration (51, 53). According to a study conducted on patients in Iran, 5.4% of people hospitalized due to Covid-19 had hypothyroidism. Most participants were over 50 years old and did not have a higher mortality rate than those without hypothyroidism (54). In the THYRCOV study (39), only 5.2% of 287 patients admitted to an intensive care unit had hypothyroidism. The mortality rate of hospitalized patients with TSH concentration above the reference range was higher than that of patients within the normal range. However, the duration of hospitalization was similar for the two groups.

Nevertheless, another study showed that hypothyroidism does not affect the outcome of the disease (50). In a retrospective study conducted in New York City, the role of hypothyroidism as a possible risk factor for poor prognosis in patients with COVID-19 was further investigated. Evidence of a specific relationship exists between lung injury associated with COVID-19 and thyroid (55). In a study by Gerwen et al., patients with COVID-19 had 251 (6.8%) of 3,703 COVID-19 patients had pre-existing hypothyroidism, and pre-existing hypothyroidism was associated with hypothyroidism. It significantly affected the patient’s adverse outcome, such as the risk of hospitalization and death. However, another study found that hypothyroidism did not predispose to the prognosis of COVID-19, including the risks of hospitalization, mechanical ventilation, and death (56), according to Liu et al. (49) patients with COVID-19 with thyrotoxicosis have a higher in-hospital mortality rate and stay in the hospital longer than COVID-19 patients with normal thyroid function. In different studies on patients hospitalized due to COVID-19, ESS was presented in about 30% of patients (up to 64%) and was associated with severity, longer hospitalization, and mortality (11, 57), contrary to the results of the present study and two previous studies. Studies in the United States (55) and Iran (54) show that hypothyroid patients with COVID-19 do not have increased hospitalizations or mortality. Current literature on COVID-19 patients with thyroid dysfunction provides further evidence that targets of SARS-CoV-2 damage could originate from the thyroid gland and the entire hypothalamic-pituitary-thyroid (HPT) axis, as hypothyroidism, non-thyroid, and thyrotoxic diseases are indicated.

Limitations of our study include: 1- Even though our study had sufficient power to detect significant differences in mortality between groups, it may have had selection bias because it was a retrospective, single-center study. 2- Because of the retrospective nature of the study, it was not possible to evaluate thyroid function using laboratory tests. 3- Due to the lack of follow-up after discharge, we could not analyze the progress of thyroid hormones over time. 4- Due to the study’s cross-sectional nature, we could not conclude whether thyroid involvement can affect the severity of COVID-19 or whether COVID-19 may cause thyroid involvement in patients.

## Conclusion

In general, the results of our study showed that thyroid involvement was related to adverse outcomes (increased mortality and increased hospitalization in the ICU). Also, thyroid involvement was related to the severity of COVID-19 disease, so most patients with thyroid gland involvement had severe and very severe lung involvement. Therefore, thyroid function monitoring should be strengthened in patients with COVID-19.

## Data availability statement

The data that support the findings of this study are available on request from the corresponding author.

## Ethics statement

This research was approved by the Faculty Research Ethics Committee of Shiraz University of Medical Sciences with the Ethical Approval Code of IR.SUMS.REC1400.306.

## Author contributions

FY: Writing – review & editing, Visualization, Validation, Resources, Project administration, Investigation. SS: Writing – original draft, Investigation, Data curation, Conceptualization. BZ-R: Writing – review & editing, Validation, Supervision, Methodology, Investigation, Formal analysis, Data curation. SR: Writing – original draft, Visualization, Resources, Investigation. MS-M: Writing – review & editing, Methodology, Investigation, Data curation, Conceptualization.
